# Sex/Gender and Socioeconomic Differences in the Predictive Ability of Self-Rated Health for Mortality

**DOI:** 10.1371/journal.pone.0030179

**Published:** 2012-01-19

**Authors:** Akihiro Nishi, Ichiro Kawachi, Kokoro Shirai, Hiroshi Hirai, Seungwon Jeong, Katsunori Kondo

**Affiliations:** 1 Department of Society, Human Development, and Health, Harvard School of Public Health, Boston, Massachusetts, United States of America; 2 Institute for Quantitative Social Science, Harvard University, Boston, Massachusetts, United States of America; 3 Department of Public Health, Graduate School of Medicine, The University of Tokyo, Tokyo, Japan; 4 Department of Global Health and Population, Harvard School of Public Health, Boston, Massachusetts, United States of America; 5 Department of Human Sciences, School of Law and Letters, University of the Ryukyus, Okinawa, Japan; 6 Iwate University Faculty of Engineering, Iwate University, Morioka, Japan; 7 Center for Well-being and Society, Nihon Fukushi University, Nagoya, Japan; Bremen Institute of Preventive Research and Social Medicine, Germany

## Abstract

**Background:**

Studies have reported that the predictive ability of self-rated health (SRH) for mortality varies by sex/gender and socioeconomic group. The purpose of this study is to evaluate this relationship in Japan and explore the potential reasons for differences between the groups.

**Methodology/Principal Findings:**

The analyses in the study were based on the Aichi Gerontological Evaluation Study's (AGES) 2003 Cohort Study in Chita Peninsula, Japan, which followed the four-year survival status of 14,668 community-dwelling people who were at least 65 years old at the start of the study. We first examined sex/gender and education-level differences in association with fair/poor SRH. We then estimated the sex/gender- and education-specific hazard ratios (HRs) of mortality associated with lower SRH using Cox models. Control variables, including health behaviors (smoking and drinking), symptoms of depression, and chronic co-morbid conditions, were added to sequential regression models. The results showed men and women reported a similar prevalence of lower SRH. However, lower SRH was a stronger predictor of mortality in men (HR = 2.44 [95% confidence interval (CI): 2.14–2.80]) than in women (HR = 1.88 [95% CI: 1.44–2.47]; p for sex/gender interaction = 0.018). The sex/gender difference in the predictive ability of SRH was progressively attenuated with the additional introduction of other co-morbid conditions. The predictive ability among individuals with high school education (HR = 2.39 [95% CI: 1.74–3.30]) was similar to that among individuals with less than a high school education (HR = 2.14 [95% CI: 1.83–2.50]; p for education interaction = 0.549).

**Conclusions:**

The sex/gender difference in the predictive ability of SRH for mortality among this elderly Japanese population may be explained by male/female differences in what goes into an individual's assessment of their SRH, with males apparently weighting depressive symptoms more than females.

## Introduction

The single-item measure of self-rated health (SRH) (“In general, how do you rate your overall health? Excellent, good, fair, or poor?”) is perhaps the most widely adopted health-status assessment approach in studies around the world [1]–[3]. Among the reasons for its popularity are its brevity, test-retest reliability, and criterion validity (i.e., its ability to predict subsequent mortality from the view that mortality is the most “objective” measure of “true” health) [4]–[8].

More recently, however, studies have begun to focus on the differential performance of the predictive ability of SRH for mortality across population subgroups [3], [6], [9]–[19]. For example, some studies have found that the ability of SRH to predict subsequent mortality is higher among more educated individuals compared to those with fewer years of schooling [10], [18], although not all studies have reported this result. In addition, some studies have found the predictive ability of SRH for mortality to be higher among men than women [9], [10], [14]–[16], [19].

There are alternative explanations for the differential performance of SRH by population subgroups [18], [20]. First, some groups may be more attuned to their health status and, thus, able to provide a more accurate, condensed assessment of their bodily conditions. For example, if less educated individuals inaccurately assess their health status (i.e., either subjectively over- or under-rating their “true” health status), then such non-differential exposure misclassifications will tend to attenuate the association of SRH with the health outcome (mortality). Alternatively, different population subgroups may be systematically biased in their subjective assessments. For example, if women are overly sensitive to their somatic symptoms and exaggerate their health problems — while, conversely, men deny and downplay their problems if they are not severe or life-threatening — then one would expect to see a stronger association between SRH and mortality among men. Spiers and her colleagues suggest that the sex/gender difference in the SRH-mortality relationship is due to “variation in the definitions that individuals call upon when rating their health,” rather than to actual differences in physical conditions [14].

In short, researchers need to have a better understanding of the sources of the differential performance of SRH in tapping the underlying health status that they are trying to capture. In the present study, we specifically focus on examining sex/gender and education-level differences in the relationship between SRH and mortality and on testing whether the differences in these associations could be explained by underlying variations in (objectively assessed) health conditions.

Based on previous reports in Western settings [18], [19], we hypothesized that we would find (1) a weaker association between SRH and mortality among women compared to men and that this finding could be explained by a stronger link between SRH and non-life-threatening physical conditions among women, and (2) a stronger association between SRH and mortality among individuals with higher education levels.

## Methods

### Participants

The analyses in the study were based on an observational prospective study, “AGES 2003 Cohort Study,” which is part of the larger Aichi Gerontological Evaluation Study (AGES). Details of this project have been described elsewhere [21], [22]. Briefly, we first obtained approval for our study in six municipalities in Chita Peninsula (south of Nagoya, the fourth largest city in Japan), Aichi Prefecture. Using a cluster random sampling approach, we sampled people from the municipalities who were at least 65 years old and did not need nursing care in 2003. A self-administered questionnaire was mailed to eligible individuals in late 2003, and 14,804 individuals returned the questionnaire. The enrollment rate was 50.4%, which is quite favorable compared to other cohort studies (for example, the Nurses' Health Study II had a baseline participation rate of around 24%) (see official website of Nurses' Health Study – accessed at http://www.channing.harvard.edu/nhs/?page_id=70). According to the limited information available on non-respondents, which was provided from several of the six municipalities, there were no large differences between respondents and non-respondents in terms of age and gender, while people with a higher socioeconomic status were more likely to respond (65.1% for the highest socioeconomic status group; 49.5% for the lowest socioeconomic status group) [22]. The study protocol and informed consent procedure were approved by the ethics committee in Research of Human Subjects at Nihon Fukushi University. The vital status of subjects enrolled in the AGES 2003 cohort was obtained by matching them to the residential basic database in local governments. We recorded subjects' date of death and any moves out of the residential area. After eliminating these people, we followed up on the remaining 14,668 subjects for the next 1,461 days (48 months).

### Measures

**Self-rated health:** Using the 2003 baseline questionnaire, we assessed SRH using the question, “How would you rate your overall health at the present time?” Four response options were provided: excellent, good, fair, or poor. On the questionnaire, this item was the third question (following inquiries about the subject's sex/gender and age) to avoid anchoring or priming the question by other questions related to health conditions (which were asked about in later sections of the survey).

**Co-morbidities:** The same baseline self-administered questionnaire was used to measure other health conditions by asking whether individuals were currently receiving medical attention for a variety of specific life-threatening physical conditions, including cancer, heart disease, stroke, hypertension, diabetes mellitus, hyperlipidemia, respiratory disease, or liver disease (respondents answered yes or no to each). We additionally assessed daily living activities (walking, bathing, and hygiene), as well as 10 non-life-threatening physical conditions (obesity, osteoporosis, joint disease/neurological pain, trauma/fracture, gastrointestinal disease, visual impairment, hearing impairment, urinary disorder, sleep disorder, and other). These distinctions were based on a previous study conducted in the Netherlands on the same issue [10]. Subjects' mental-health status was assessed by the Geriatric Depression Scale (GDS-15) [23]. Health-related behaviors (smoking [never, before, currently] and alcohol consumption [no, not frequently, frequently]) were also measured.

**Socio-demographic characteristics:** We included the following socio-demographic characteristics in the study: age, sex/gender, education (<high school [<10 years of education], high school [10–12 years of education], and >high school [>12 years of education]), and marital status (ever married or never married).

**Statistical analysis:** We created logistic regression models to explore the associations between SRH and sex/gender and education. Education (<high school or not) and SRH (fair/poor or not) were dichotomized to allow us to apply a concise interpretation of the interaction (product term). We conducted the following sequence of regression analyses (Models 1A to 5A): in Model 1A, we controlled for age only. In Model 2A, we controlled for marital status and whether the subject smoke or drank, in addition to age. In Model 3A, we added depression symptoms. In Model 4A, we added 13 non-life-threatening health conditions. Finally, in Model 5A, we added eight major life-threatening health conditions.

To explore the SRH-mortality association, Cox models were used to estimate hazard ratios (HRs) for mortality in the different sex/gender and education-level subgroups. The proportionality of all predictors was examined graphically beforehand using log-minus-log survival plots. We then created Models 1B to 4B, in which we added SRH as an exposure variable to Models 1A to 4A.

In both the logistic regression and Cox models, we added interaction terms (male×<high school, fair/poor SRH×male, and fair/poor SRH×<high school) to the sets of explanatory covariates to statistically test the marginal difference in the effect size in the different sex/gender and education-level subgroups.

The relative risk reduction (i.e., explainable excessive risk) resulting from the successive addition of covariates from HR Model xB to HR Model (x+1)B was calculated as follows, where x = 1, 2, 3, or 4 [24], [25]:
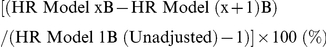
The assumption is that the risk reduction calculated from HR Model xB to HR Model (x+1)B can be interpreted as the contribution of the added covariates to the excess risk for mortality associated with fair/poor SRH.

Clustered standard error was calculated in all of the analyses to account for clustering of respondents in the six sampled municipalities [26]. A two-tailed p value of <0.05 was considered statistically significant. We used Stata/IC version 12.0 (StataCorp, College Station, TX, USA).

## Results

### Sex/gender-SRH and education-SRH associations at baseline

As expected, increasing age was associated with higher levels of depression, an increase in non-life-threatening health conditions, and a higher prevalence of fair/poor SRH among both men and women (**Table 1**). Higher levels of depression and non-life-threatening health conditions were more prevalent among women than men. Lower educational attainment (<high school) was also associated with a higher prevalence of fair/poor SRH (p<0.001 among both sexes/genders; the p value was obtained using the age-adjusted logistic regression model for the education-SRH association) (**Table 2** **[Model 1A]**).

**Table 1:** Self-rated health at baseline and four-year mortality in AGES 2003 Cohort Study in Japan.

			*Depression [GDS15]*		*Non-life-threatening physical conditions*		*Life-threatening physical conditions*		*Baseline self-rated health*		*Four-year Mortality*	
	*A Age*	n	Score	SE	Number	SE	Number	SE	Excellent /good	Fair/poor	Alive	Dead
**Men**	**65–69**	2525	3.28	0.13	0.61	0.03	0.76	0.02	76.2%	23.8%	94.1%	5.9%
	**70–74**	2085	3.55	0.08	0.74	0.03	0.82	0.01	73.2%	26.8%	91.4%	8.6%
	**75–79**	1368	3.83	0.17	0.90	0.03	0.88	0.01	64.7%	35.3%	85.0%	15.0%
	**80–84**	580	3.94	0.21	1.02	0.02	0.83	0.03	67.6%	32.4%	77.3%	22.7%
	**85–**	251	4.25	0.37	1.28	0.09	0.71	0.03	66.9%	33.1%	61.4%	38.6%
**Women**	**65–69**	2374	3.38	0.09	0.69	0.02	0.65	0.02	78.0%	22.0%	97.8%	2.2%
	**70–74**	2089	3.81	0.06	0.94	0.03	0.76	0.02	70.9%	29.1%	96.4%	3.6%
	**75–79**	1658	4.13	0.14	1.17	0.01	0.80	0.01	64.2%	35.8%	94.4%	5.6%
	**80–84**	824	4.27	0.20	1.19	0.08	0.79	0.03	66.3%	33.7%	90.8%	9.2%
	**85–**	424	4.79	0.13	1.25	0.05	0.71	0.03	68.9%	31.1%	73.1%	26.9%
	***B Education***											
**Men**	**<High school**	3801	3.97	0.09	0.83	0.03	0.78	0.01	68.7%	31.3%	87.5%	12.5%
	**High school**	1822	2.97	0.10	0.66	0.03	0.83	0.02	76.7%	23.3%	90.7%	9.3%
	**>High school**	907	3.00	0.12	0.71	0.03	0.88	0.02	75.5%	24.5%	91.3%	8.7%
**Women**	**<High school**	4633	4.11	0.08	0.99	0.01	0.72	0.01	69.1%	30.9%	94.0%	6.0%
	**High school**	2120	3.31	0.09	0.92	0.03	0.77	0.02	75.2%	24.8%	95.1%	5.0%
	**>High school**	370	3.17	0.12	0.83	0.03	0.75	0.04	77.5%	22.5%	96.0%	4.1%

**Table 2:** Odds ratios of sex/gender and education for fair/poor self-rated health at baseline in logistic regression models^a^.

	Model 1A^b^			Model 2A^b^			Model 3A^b^			Model 4A^b^			Model 5A^b^		
	(Adjusted for age)			(Model 1A+marital status, smoking, and drinking)			(Model 2A+depression [GDS15])			(Model 3A+13 non-life-threatening physical conditions)			(Model 4A+8 life-threatening physical conditions)		
	ORs	95%CI		ORs	95%CI		ORs	95%CI		ORs	95%CI		ORs	95%CI	
**Sex/gender (Male vs female)**	**0.99**	0.86	1.14	**1.09**	0.89	1.35	**1.17**	0.97	1.41	**1.27**	1.09	1.48	**1.14**	0.99	1.31
**Education (<high school vs high/>high school)**	**1.38**	1.30	1.47	**1.36**	1.26	1.46	**1.11**	1.00	1.23	**1.08**	0.98	1.19	**1.16**	1.07	1.26
***Interaction term (male*** **×** ***<high school)***	p = 0.613			p = 0.819			p = 0.861			p = 0.940			p = 0.818		

a Interaction terms were excluded in the models when we reported the ORs and 95% CIs on the table (effect-only models).

b OR of sex/gender and that of education were calculated by different two models in Model 1A. In contrast, OR of sex/gender and that of education were calculated simultaneously by a single model in Model 2A, 3A, 4A, and 5A.

In the age-adjusted model (**Table 2**), men were no more likely to report fair/poor health than women. Subsequent models that adjusted for sex/gender differences in pre-existing co-morbid conditions resulted in a higher odds ratio for poor SRH among men compared to women. For example, after controlling for 13 co-morbid conditions, men were 1.27 times more likely to report poor SRH than women (95% CI: 1.09–1.48) (**Table 2** **[Model 4A]**).

In the age-adjusted model, individuals who had completed less than a high school education were 1.38 times more likely to report fair/poor health compared to individuals with higher levels of completed schooling (95% CI: 1.30–1.47). However, after adjusting for differences in chronic health conditions, this excess risk was attenuated. This suggests that individuals with less education have more co-morbid chronic conditions, which is identical to the observation in **Table 1**.

### SRH-mortality associations

During the four-year follow-up (from 2003 to 2007), 788 men died in 26,482.9 person-years, and 430 women died in 29,723.6 person-years. Additionally, 58 men and 111 women moved out of the study area. Older individuals, men, and individuals with a lower education level had higher mortality rates (**Table 1**).

Cox models for the SRH-mortality association (**Table 3-I**) showed that individuals who reported fair/poor SRH were approximately two times more likely to die during the follow-up period (age-adjusted HR = 2.20 [95% CI: 1.87–2.58], without including interaction terms). In the same model, the interaction for sex/gender was statistically significant (p = 0.018), suggesting men exhibit a stronger association between SRH and mortality than women. The interaction for education was not significant (p = 0.549).

**Table 3:** Hazard ratios of self-rated health (SRH), sex/gender, and education for mortality during four-year follow-up in Cox models^a^.

	Model 1B^b^			Model 2B^b^			Model 3B^b^			Model 4B^b^			Model 5B^b^		
	(Adjusted for age)			(Model 1B+marital status, smoking, and drinking)			(Model 2B+depression [GDS15])			(Model 3B+13 non-life-threatening physical conditions)			(Model 4B+8 life-threatening physical conditions)		
***I Both sex/genders***															
***Both sex/genders with all education levels***	**HRs**	**95% CI**		**HRs**	**95% CI**		**HRs**	**95% CI**		**HRs**	**95% CI**		**HRs**	**95% CI**	
**SRH (fair/poor vs excellent/good)**	**2.20**	1.87	2.58	**2.23**	1.90	2.61	**1.97**	1.70	2.28	**1.97**	1.67	2.31	**1.67**	1.35	2.07
**Sex/gender (Male vs female)**	**2.06**	1.78	2.39	**2.06**	1.57	2.71	**2.06**	1.55	2.74	**1.99**	1.48	2.67	**1.90**	1.38	2.62
**Education (<high school vs high/>high school)**	**1.12**	0.90	1.38	**1.05**	0.87	1.27	**0.96**	0.79	1.16	**0.95**	0.78	1.15	**0.93**	0.79	1.11
**Depression (GDS15)** c	**1.08**	1.07	1.11	**-**			**1.05**	1.03	1.07	**1.04**	1.02	1.06	**1.04**	1.02	1.06
***Interaction term (fair/poor SRH***×***male)***	p = 0.018			p = 0.090			p = 0.465			p = 0.449			p = 0.924		
***Interaction term (fair/poor SRH***×***<high school)***	p = 0.549			p = 0.364			p = 0.221			p = 0.216			p = 0.258		
***II Stratified by Sex/gender***															
***Men with all education levels***															
**SRH (fair/poor vs excellent/good)**	**2.44**	2.14	2.80	**2.36**	2.00	2.78	**2.00**	1.71	2.34	**2.05**	1.69	2.48	**1.70**	1.35	2.15
**Education (<high school vs high/>high school)**	**1.19**	0.90	1.56	**1.05**	0.84	1.32	**0.93**	0.73	1.19	**0.93**	0.71	1.23	**0.91**	0.72	1.15
***Women with all education levels***															
**SRH (fair/poor vs excellent/good)**	**1.88**	1.44	2.47	**2.01**	1.66	2.43	**1.93**	1.55	2.40	**1.85**	1.40	2.43	**1.67**	1.16	2.42
**Education (<high school vs high/>high school)**	**1.11**	0.87	1.42	**1.06**	0.75	1.48	**1.02**	0.75	1.39	**0.97**	0.71	1.33	**0.97**	0.71	1.33
***III Stratified by Education***															
***Lower education (both sex/genders)***															
**SRH (fair/poor vs excellent/good)**	**2.14**	1.83	2.50	**2.15**	1.83	2.52	**1.86**	1.50	2.30	**1.85**	1.53	2.23	**1.61**	1.21	2.14
**Sex/gender (Male vs female)**	**2.40**	2.01	2.89	**1.96**	1.36	2.82	**1.89**	1.29	2.75	**1.83**	1.27	2.65	**1.77**	1.18	2.65
***Higher education (both sex/genders)***															
**SRH (fair/poor vs excellent/good)**	**2.39**	1.74	3.30	**2.43**	1.85	3.19	**2.24**	1.59	3.16	**2.25**	1.56	3.24	**1.74**	1.20	2.52
**Sex/gender (Male vs female)**	**2.28**	1.79	2.90	**2.27**	1.44	3.58	**2.40**	1.52	3.79	**2.36**	1.41	3.95	**2.20**	1.34	3.63

a Interaction terms were excluded in the models when we reported the HRs and 95% CIs on the table (effect-only models).

b HR of each cell in Model 1B was calculated by each different model. In contrast, HR of sex/gender and that of education (and that of depression after Model 3B) were calculated simultaneously by a single model in Model 2B, 3B, 4B, and 5B.

c As depression was included as the control variable in Model 3B in addition to the variables in Model 2B, HR of depression in Model 2B was not reported.

The stratified analyses by sex/gender (**Table 3-II**) showed that the SRH-mortality association among men (age-adjusted HR = 2.44 [95% CI: 2.14–2.80]) was stronger than the corresponding association among women (age-adjusted HR = 1.88 [95% CI: 1.44–2.47]). The stratified analyses by education level (**Table 3-III**) also showed that the SRH-mortality association among people with a higher education (age-adjusted HR = 2.39 [95% CI: 1.74–3.30]) seemed to be stronger than among those with a lower education (age-adjusted HR = 2.14 [95% CI: 1.83–2.50]). (The interaction terms shown were not significant).

When we examined the regression models with the successive introduction of control variables, the sex/gender difference in the association of fair/poor SRH and mortality was equalized after we controlled for the complete set of co-morbid conditions (Model 5B male HR = 1.70; Model 5B female HR = 1.67).

**Figure 1:**
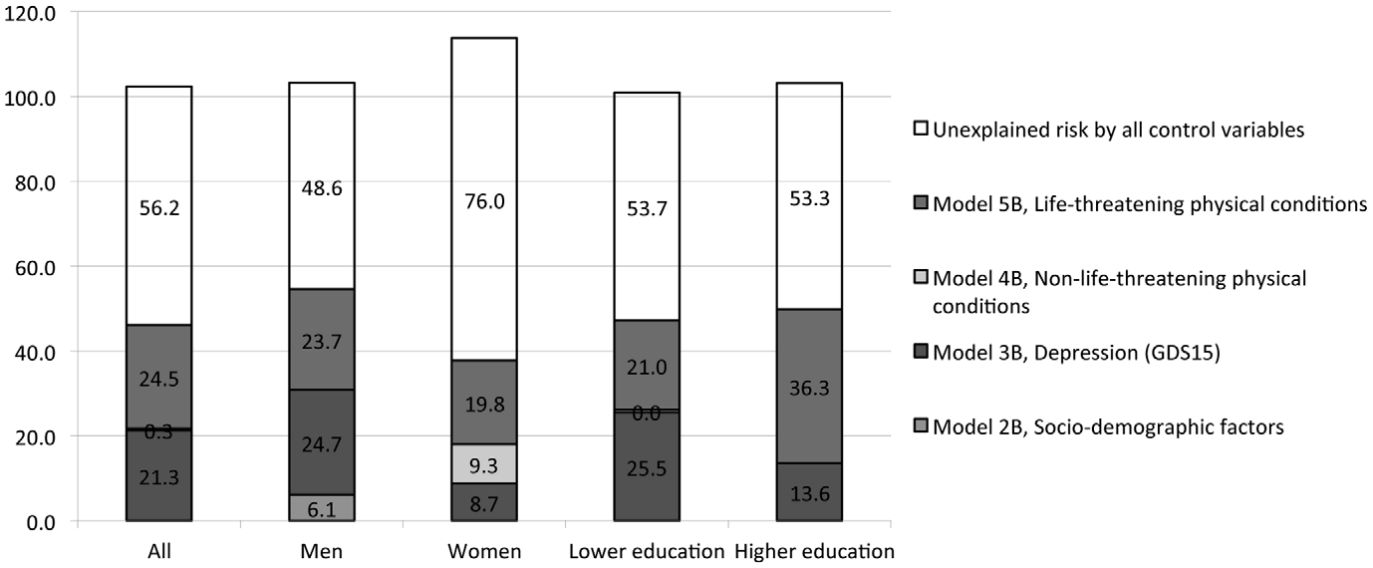
Percentages of explainable excessive risk of self-rated health for age-adjusted mortality by other self-reported measures added in Model 1B to 5B^a^. ^a^ As some values of the explainable excessive risk by other self-reported measures (relative risk reduction) were negative (i.e., −X.X%), the sum of the percentages in each bar was not 100% among several subgroups.

We next examined the contributions of different clusters of covariates to the excess risk linking fair/poor SRH to mortality. We illustrate this for the overall sample, as well as for men versus women and low versus high education levels (**Figure 1**). Among men, 48.6% of the excess risk could not be explained by the variables in our models, whereas 76.0% of the excess risk among women could not be explained.

Depression symptoms explained 24.7% of the excess risk of fair/poor SRH for mortality for men, as compared to 8.7% for women. Depression symptoms also explained 25.5% of the excess risk of fair/poor SRH for mortality among people with lower education levels, as compared to 13.6% among those with a higher education.

## Discussion

Several noteworthy findings emerged regarding sex/gender and socioeconomic differences in the SRH-mortality association among this Japanese older population, as compared with previous studies from other countries. Our first hypothesis (stronger association among men) was partially supported by the analysis. While a stronger association was found among men, we found this was because the presence of depressive symptoms had a stronger influence on men's ratings of SRH than women's ratings. This was contrary to our prior hypothesis that the presence of physical health conditions would explain the stronger association of SRH to mortality among men.

Our second hypothesis (stronger association between SRH and mortality among more educated individuals) was not supported by our analyses. The predictive ability of SRH for mortality was similar among individuals with different levels of schooling.

### Comparisons with previous studies

We found three noteworthy results with respect to the sex/gender difference in SRH. First, Japanese women and men report roughly the same prevalence of fair/poor SRH. This is consistent with the findings of some previous studies in Western settings, where women live longer than men but also tend to report poorer SRH [9], [19]. In our study, older Japanese men were more likely to report fair/poor SRH than women after we controlled for sex/gender differences in the prevalence of additional self-reported co-morbid health conditions. Our second noteworthy finding is that the association between fair/poor SRH and subsequent mortality was stronger for men than women. This finding is consistent with other studies from the Netherlands [19] and the United Kingdom [14]. Third, the sex/gender difference in the association of fair/poor SRH and mortality was progressively attenuated (and almost eliminated) after controlling for additional self-reported health conditions. Our findings in this regard are inconsistent with previous studies from the Netherlands [19] and the United Kingdom [14], both of which used almost the same statistical procedures and reported that the sex/gender differential in the predictive ability of SRH remained even after controlling for a range of health conditions.

In terms of differences associated with educational attainment, our study made four noteworthy findings. First, people with lower educational attainment were more likely to report poor SRH, which was attenuated (or explained) when we added co-morbid conditions to the regression models. This implies that differences in SRH across education-level groups reflect the real underlying variation in “objective” health. Second, we found that the SRH-mortality association tended to be similar across education levels. This finding is consistent with previous studies reported in Sweden [17] and the United Kingdom [12] but inconsistent with studies from the United States [18] and the Netherlands [10]. We used educational attainment as the proxy measure of relative socioeconomic status, and differences in the predictive ability of SRH according to socioeconomic status could be related to how egalitarian a country's society is (see the GINI coefficient of each country among 1992–2007, Japan: 0.25, Sweden: 0.25, United Kingdom: 0.36, United States: 0.41, and Netherland: 0.31) [27]. Our interpretation of this difference based on education level is that although people with less education have more health problems (and hence report poorer overall SRH), their assessments of their health are less accurate than those of people with more education in terms of rating the impact of potentially life-threatening conditions on their SRH levels. Third, we found that the non-significant educational difference in the predictive ability of poor SRH was more attenuated when we added life-threatening physical conditions as control variables. Lastly, we found that depression symptoms explained about 26% of the excess risk of poor SRH on mortality among individuals with a lower education level, while life-threatening physical conditions played a greater role (36%) in explaining excess risk among individuals with a higher education level.

**How do people assess their own health?** A recent landmark review article on SRH provided a theoretical framework for how people assess their own health [3], [28]. According to Jylha's theory, people go through three stages in the process of assessing their health: (1) “recognizing the meaning of health and identifying the components that should be included as components of self health,” (2) “considering the way in which those components should be taken into account,” and (3) “deciding which of the levels in the presented scale best summarizes these components.” Thus the theory posits that individuals move through a logical and sequential series of mental steps when asked to rate their health status. . However, the logical flow of Jylha's theory has been criticized because (1) an individual's decision-making process is not that logical and is influenced by psychological filters and (2) each individual does not have access to their complete health information for the decision-making process [28]. Therefore, critics argue that Jylha's assumption (i.e., that individuals are rational enough to thoroughly and carefully think through the three stages one by one) does not fit with reality and that individuals are more heuristic [28], [29].

Our research findings can contribute to the theoretical debate on at least three points. First, people seem to rate their own health in a heuristic manner, as proposed by Huisman and Deeg [28], because the study participants in every subgroup exhibited an excess risk for mortality which could not be completely explained by a set of diagnosed illnesses and socio-demographic factors. Second, the percentages of explainable excessive risk varied according to socio-demographic factors and depression symptoms, and moreover, the set of explanatory factors differed for each subgroup. This suggests that people in different subgroups may be differentially utilizing information in order to rate their health. Third, when we focus on the sex/gender difference, the percentage of unexplainable excessive risk was greater among women (76%) than men (48.6%), implying that Japanese women are more heuristic than Japanese men in the process of assessing their own health (assuming that added control variables constitute the available information for assessing their own health). Overall, our findings support the argument that a framework of psychological factors should be added to Jylha's theoretical framework, as Huisman and Deeg suggest [3], [28].

**What is SRH?** Beyond the debate on how people assess their own health, a more fundamental set of questions raised by this research include: What is SRH? Subjective health? Objective health? True health? Although these questions have not yet been answered, several scholars have suggested measuring true/objective health using the SRH question in self-administered questionnaires via several methods, including Jylha's theoretical framework [3], described above; an anchoring vignette [2]; and validity evaluation with bio-markers [3], [30], where the focus in relation to SRH is the distance between latent true/objective health and self-rated subjective health [20], [30]. On the other hand, several scholars discuss how SRH is “a measure of people's perception of their health rather than a measure of true health,” and therefore, it can be “the most informative from the holistic point of view” [28].

Although our findings cannot provide us with a direct clue about what SRH is, the stepwise inclusion of control variables in adjusted models is suggestive of what “goes into” an individual's assessment of SRH. However, two important hypothetical explanations are missing from Jylha's theory and the foregoing discussion, which should be discussed here to understand our findings more deeply [3]. First, bio-physiological changes inside the body, which can be detected by slight changes in the level of a bio-marker (inflammatory, immunological, endocrinological, etc.) through a blood test and which the host (the individual) has not yet perceived in their mind, can contribute to SRH. Several bio-markers (e.g., hemoglobin, albumin, interleukin-1 β, tumor necrosis factor α) have been associated with SRH, and such factors can contribute to the host's SRH without their knowledge [3], [30]. Thus, the single-item SRH question and its answer may be partly based on the rich information provided by ongoing bio-physiological changes in the body, which can be useful in the context of promoting activities and preventive medicine.

Second, the distance or discrepancy between subjective health and objective health cannot be merely a measurement error; it is a causal factor (beyond a predictive factor) for mortality. SRH is a form of self-fulfilling prophecy [31]. This idea is captured in a traditional Japanese proverb, “*yamai wa ki kara*,” which translates as “illness springs from one's spirit” (“*ki*”) and refers to the Japanese conception that physical illness can result from a person's frame of mind toward body and physical illness itself [32]. It is possible that individuals who report lower SRH feel defeated in some way and that this state of mind has an adverse effect on their physical health. To date, no study has been able to tease this out.

Therefore, future studies need to work on testing the “self-fulfilling prophecy” hypothesis for the SRH-mortality association, as well as establishing the theory of assessing one's health to see the bio-physiological mechanism behind the SRH-mortality association. To establish the model, numerous broad scientific studies — from molecular to social — are required now. Overall, the theory of assessing one's health could incorporate psychological filters, not-yet-perceived bio-physiological changes, and self-fulfilling prophecy into one explanatory model.

### Study limitations

There are several limitations of and points of discussions regarding the present study. First, the duration of follow-up (four years) was relatively short compared to some previous studies (up to 10 years) [6]. Although this limitation could be overcome with additional follow-up with the same cohort, the trade-off is changes in SRH (and hence exposure to misclassification) over time. Second, the relatively low response rate to the baseline questionnaire (50.4%) could yield a risk of selection bias (e.g., biased estimate of HR of SRH for mortality). However, the differences in the socioeconomic status (see the Introduction to this paper) and health condition characteristics (unknown and not reported, but possible) between respondents and non-respondents were arguably not likely to make the estimate biased because such differences could not directly affect the SRH-mortality association itself, at least not after the adjustment for education and health conditions (life-threatening and non-life-threatening diseases). If more information on non-respondents was available, we could test the explanation above using statistical analysis or perform the regression analysis with multiple imputation technique. Third, educational attainment levels among the older study population could differ from that of the entire Japanese population, given the rapid economic development of Japan over the last 50 years, which has led to an increase in educational attainment with each birth cohort.

### Conclusions

In summary, we did not find a socioeconomic difference in the SRH-mortality association, but we did find a sex/gender difference, which was attenuated after adjusting for several social and medical factors. Although the theoretical framework provided by Jylha [3] hinted at this sex/gender difference, biological and psychological factors may need to be incorporated into the model.
